# Prevalence of Anemia and Associated Factors among Infants and Young Children Aged 6–23 Months in Debre Berhan Town, North Shewa, Ethiopia

**DOI:** 10.1155/2020/2956129

**Published:** 2020-12-17

**Authors:** Abebaw Molla, Gudina Egata, Firehiwot Mesfin, Mikyas Arega, Lemma Getacher

**Affiliations:** ^1^School of Public Health, College of Medicine and Health Sciences, Mizan-Tepi University, Mizan Teferi, Ethiopia; ^2^School of Public Health, College of Health and Medical Sciences, Haramaya University, Harar, Ethiopia; ^3^School of Nursing and Midwifery, College of Health and Medical Sciences, Haramaya University, Harar, Ethiopia; ^4^Department of Midwifery, College of Medicine and Health Sciences, Debre Berhan University, Debre Berhan, Ethiopia; ^5^Department of Public Health, College of Medicine and Health Sciences, Debre Berhan University, Debre Berhan, Ethiopia

## Abstract

**Background:**

Anemia is a problem of both the developed and developing world, which occurs in all age groups of the population. Half of the anemia cases are due to iron deficiency and affects physical growth and mental development. Nevertheless, there is a scarcity of information about anemia and associated factors among infants and young children aged 6 to 23 months in low-income countries like Ethiopia.

**Objective:**

The aim of this study was to assess the prevalence of anemia and associated factors among infants and young children aged 6–23 months.

**Methods:**

A community-based cross-sectional study design was used among 531 mothers/caregivers-children pairs in Debre Berhan Town, North Shewa, Ethiopia, from February 1 to March 2, 2018. The cluster sampling technique was used to select the study participants. Sociodemographic data were collected from mothers/caregivers using pretested structured questionnaires. Hemoglobin levels were measured using a HemoCue analyzer machine (HemoCue® Hb 301, Ängelholm, Sweden). All relevant data were described using descriptive statistics such as frequencies, proportions, mean, and standard deviation. Odds ratio and 95% CI were estimated using binary logistic regression to measure the strength of the association between anemia and explanatory variables. The level of statistical significance was declared at *P* < 0.05.

**Results:**

The overall prevalence of anemia was 47.5% (95% CI: 43.1–51.4%) of which 18.3% were mildly anemic, 25% were moderately anemic, and 4.1% were severely anemic. In multivariable logistic regression analysis, household food insecurity (AOR = 2.7, 95% CI: 1.6–4.5), unmet minimum dietary diversity (AOR = 2.5, 95% CI: 1.4–4.3), stunting (AOR = 2.3, 95% CI: 1.2–4.3), and underweight (AOR = 2.7, 95% CI: 1.4–5.4) positively associated with anemia while having ≥4 antenatal care visits (AOR = 0.5, 95% CI: 0.3–0.9) and met minimum meal frequency (AOR = 0.25, 95% CI: 0.14–0.45) had a protective effect against anemia.

**Conclusion:**

Generally, the study showed that anemia was a severe public health problem among infants and young children in the study setting. Antenatal care visit, meal frequency, dietary diversity, underweight, stunting, and food insecurity significantly associated with anemia. Therefore, efforts should be made to strengthen infant and young child feeding practices and antenatal care utilization and ensure household food security, thereby improving the nutritional status of children.

## 1. Introduction

Nutrition during the first 1000 days is very critical for child health, growth, cognitive development, and productivity in later life [1]. Children have rapid physical growth, mental development, and the highest nutritional demand in the first two years of life. This period of life is coupled with the introduction of complementary foods, which are low in their iron content and can result in anemia. Thus, inadequate nutrition leaves short-term and long-term irreversible effects on physical growth and mental development [2, 3].

Anemia is defined as a low hemoglobin concentration in the blood leading to poor oxygenation of body tissue. It occurs at all age groups at one point in the life cycle but mostly affects reproductive age women and under-five children [4, 5]. Anemia among under-five children is defined as hemoglobin value less than 110 g/L and classified as mildly anemic (10–10.9 g/dL), moderately anemic (7–9.9 g/dL), and severely anemic (<7 g/dL) [6]. Globally in 2011, 47% of children under five years of age were anemic [7]. According to the 2013 systematic analysis of anemia for 1995–2011, about 25% to 58% of under-five children in Asia and 46% to 71% of under-five children in Africa were anemic [8]. The 2016 systematic analysis of 25 sub-Saharan African countries (SSA) revealed that 71.1% of children aged 6–23 months were anemic [9]. In Ethiopia, the prevalence of anemia among under-five children is 57%, of which 72% are among infants and young children. However, the magnitude varies from region to region in the country, 42% in Amhara Region and 83% in Somali Region. Accordingly, a study done in northeast Ethiopia reported that 41.1% of under-five children were anemic [10]. In the same line, the prevalence of anemia among reproductive age women of 15–49 years showed an increase from 17% in 2011 to 24% in 2016 EDHS [11]. Similarly, studies conducted in Gondar Town, Gondar University Hospital, and Adigrat Hospital showed that the magnitude of anemia among pregnant women was 56.8%, 22.2%, and 7.9%, respectively [12–14].

Anemia reflects overall population health and nutritional status. Anemia can result in different health consequences such as maternal and child mortality, poor motor and cognitive development, high school absenteeism, poor school performance, and reduced working capacity among adults [6, 15]. Worldwide, anemia accounted for 115000 maternal and 591000 perinatal deaths. It also causes 8.8% of disability. The majority of anemia-related disabilities were found in Asia and Africa as 37.5% and 71.9%, respectively [16]. About 40% to 60% of children aged 6 to 23 months in developing countries had anemia associated cognitive development problems [17].

Anemia arises from multifaceted factors and is classified as nutritional, nonnutritional, and genetic bases [12]. Low iron content in the diet and low iron absorption are major risk factors for anemia [12, 18]. Globally, fifty percent of anemia cases are caused by iron deficiency [19], but other risk factors like micronutrient deficiencies (folic acid, zinc, and vitamin B12), parasitic infections (hookworm, schistosomiasis, ascariasis, and malaria), and blood disorders (sickle cell anemia and thalassemia) can cause anemia as well [7, 20].

Although the prevalence of anemia and associated factors among mothers and under-five children was identified in Ethiopia, it has been noticed that there could be variations across settings in the country [12–14, 21–26]. Thus, determining the prevalence of anemia and associated factors in multicultural and geographical settings and the sociodemographic context is vital to design appropriate intervention strategies that best fit the local context. Therefore, this study aimed to assess the prevalence of anemia and associated factors among infants and young children aged 6–23 months in Debre Berhan Town, Ethiopia.

## 2. Materials and Methods

### 2.1. Study Design, Setting, and Period

A community-based cross-sectional study was conducted from 1 February to 2 March, 2018, in Debre Berhan Town, North Shewa Zone, Amhara, Regional State, Ethiopia. Emperor Zara Yaqob founded the town and served as the capital of the North Shewa Zone. The town is located 130 km from Addis Ababa, the capital city of Ethiopia, and 690 km from Bahir Dar, the capital of the Amhara Region. The town has an altitude of 2840 meters above sea level. The area practices a mixed farming system: crop production with animal husbandry. The main crop production in the area is barley, wheat, peas, lentils, and linseed. Cattle, sheep, horses, donkeys, and mules are the main live stocks.

According to the 2017 Town Health Administrative Office report, the town has a total population of 88369, of whom 14011 are under-five children and 6707 are children under two years. The town has nine kebeles (the smallest administrative unit) in Ethiopia, one referral hospital, three health centers, and 14 health posts.

### 2.2. Study Participants and Sampling Procedures

The source population of the study was all infants and young children aged 6–23 months and their mothers or caregivers in Debre Berhan Town. The study population was all infants and young children aged 6–23 months and their mothers/caretakers living in three randomly selected kebeles/clusters. All mother/caregiver-child pairs living for at least six months in the study were included in the study. Mothers or caregivers who were unable to respond to the interview due to their child's or their own illness and infants and children who had taken iron or vitamin A supplements or subjected to deworming or blood loss due to injury in the past three months were excluded from the study. The cluster sampling technique was used to select mother/caregiver-child pairs. Debre Berhan Town has 9 kebeles and three randomly selected kebeles are considered as clusters. The total number of children in each selected cluster was obtained from health extension workers (HEWs) family folder documentation. Based on the records of HEWs, 577 infants and young children aged 6–23 months were found in the selected clusters.

The sample size was determined using a single population proportion formula with the following assumptions: prevalence of anemia among children aged 6–23 months to be 66.6% (22), 5% margin of error, 95% confidence level, design effect of 1.5, and 10% for nonresponse, which gave rise to 564 samples. In the case of more than one child being available in a given household, both children were included in the study. Due to the nature of cluster sampling, 577 infants and young children-mothers pairs living in selected clusters were included in the study.

### 2.3. Data Collection Methods and Instruments

Socioeconomic and demographic data of mothers or caregivers and their children were collected through home-to-home visits using a pretested structured interviewing-administered questionnaire which was adapted from similar studies [21, 22]. The birth date of the children was recorded based on mothers' or caregivers' verbal reports.

The child's dietary diversity score was assessed using the dietary diversity assessment tool adapted from the WHO standardized questionnaire for infant and young child feeding practices. It was based on the mother's or caregiver's recall of all foods given to her child in the past twenty-four hours prior to the survey. The dietary diversity score was based on seven food groups consumed by the child: grains, roots and tubers, legumes and nuts, dairy products, flesh foods, eggs, vitamin A-rich fruits and vegetables, and other fruits and vegetables [18]. Household food security status was measured using the Household Food Insecurity Access Scale (HFIAS), a structured, standardized, and validated tool developed by the Food and Nutrition Technical Assistance (FANTA), which has nine occurrences and frequency of occurrence questions based on the previous four weeks or one-month recall method [27].

Anthropometric data, such as the child's height and weight, were also collected. The child's length was measured to the nearest 0.1 centimeters using the United Nation Children's Fund (UNICEF) horizontal wooden length board with a movable headpiece on a flat surface. Children were kept in a recumbent position and the five contact points, including the head, shoulders, buttocks, calves, and heels, were maintained against the length of the board in a straight direction. The child's weight was measured to the nearest 0.1 kg. The weight of a child was estimated by subtracting the mother's/caregiver's weight record from the weight record of both mother and child obtained together. Each anthropometric measurement was measured after removing shoes, heavy clothes, and capes. Each participant was measured twice and the average value was taken when there were variations between two consecutive measurements. The weight scale was adjusted to zero level and calibrated using a standard 2 kg weight object before weighing each study participant.

Hemoglobin level was measured with a HemoCue analyzer machine (HemoCue® Hb 301, Ängelholm, Sweden). The HemoCue HB 301 analyzer has internal quality control, the self-test. Every time the analyzer is turned on, the analyzer automatically verifies the measurement performance. This test is performed at regular intervals if the analyzer remains switched on. Upon passing the self-test, the display will show the HemoCue system and three dashes showing that the analyzer is ready to perform the measurement [28]. When an error code was displayed due to self-test failure, a quality control measure was performed according to the recommended guideline.

A separate lancet was used for each child's finger pricking. After wiping off the first two drops of the blood sample, a third drop was collected and completely filled to a cuvette in one continuous motion. Hemoglobin data were adjusted during analysis at an altitude of 2840 meters above sea level and hemoglobin adjustment was done according to the WHO 2011 recommendation [29]. The hemoglobin cutoff point is based on the WHO's classification of under-five anemia, defined as hemoglobin level <11 g/dL [5]. A child with a hemoglobin value <11 g/dL was confirmed as anemic.

### 2.4. Measurements

  Anemia: In this study, anemia among children aged 6–23 months was the outcome variable, and it was understood as hemoglobin level <11 g/dL after adjustment for individual hemoglobin value by subtracting 1.9 g/dL for an altitude of 2840 meters above sea level [29].
  Food-secure household: If the household head responds no to all questions items 1–9 or responds yes to question item 1 and experiences rarely in the past four weeks [27].
  Food-insecure household: If the household head responds at least yes to question item 1 and experiences sometimes in the past four weeks [27].
  Minimum dietary diversity was achieved: The proportion of children aged 6–23 months who received 4 or more food items among the seven food groups [18].
  Minimum meal frequency was achieved: The minimum number of times a child aged 6–23 months received solid, semisolid, or soft foods. If a child is breastfed, aged 6–8 months, s/he should receive two times and if aged 9–23 months, s/he should receive three times while a nonbreastfed child aged 6–23 months should receive four times during the previous day [18].
  Stunting: Length for age <−2SD of the WHO growth standard [30].
  Underweight: Weight for age less than −2SD of the WHO growth standard [30].
  Wasting: Weight for length <−2SD of the WHO growth standard [30].
  Household wealth index: The proxy measure of household living standards based on the sum of available assets of the owner like productive assets, durable assets, domestic animals, and housing characteristics [10].
  Diarrhea: Children with a history of ≥3 times loose stool or watery diarrhea per day for two weeks prior to the study [31].
  Timely introduction of complementary feeding: The child starts to receive solid, semisolid, or soft foods at the age of six months in addition to continued breastfeeding [18].
  Early initiation of breastfeeding: The proportion of children born in the last 24 months who were put to the breast within one hour of birth [18].

### 2.5. Data Quality Control

Data collection tools were prepared in English and translated into Amharic and then translated back into English to check for its consistency. Pretest was done on 5% of the study sample in the nonselected kebele. Two days of training was given to data collectors and supervisors on the objectives and context of the study, content of the questionnaire, how to fill the questionnaire in the field, interview technique, household selection procedure, respondent approaching technique, hemoglobin, and anthropometric measurement. The relative technical error of measurement (%TEM) was calculated to minimize intra- and interobserver variability [32]. Data collection was supervised by two BSc nurses, and the principal investigator supervised the overall data collection process. Data were double-entered by two independent data clerks for cross-validation.

### 2.6. Statistical Analysis

First, data were checked for completeness, accuracy, and consistency before entering the computer. Data were then coded and entered into Epi-Data version 3.1 and exported to IBM-SPSS version 22 statistical software for analysis. The household wealth index was computed using principal component analysis (PCA) with all its assumptions, after which it was categorized into five quintiles: lowest, second, middle, fourth, and highest.

Nutritional indices of infants and young children, such as height-for-age Z-score (HAZ), weight-for-age Z-score (WAZ), and weight-for-height Z-score (WHZ), were calculated according to the WHO 2006 multicenter growth reference [23]. WHO Anthros 2005 Software version 3.2.2 was used to calculate Z-scores, and infant and young children were categorized as being stunted (HAZ <−2 SD Z-scores), underweight (WAZ <−2SD Z-scores), and wasted (WHZ <−2SD Z-scores).

Bivariate logistic regression was done to see the association between each independent variable and the outcome variable, anemia. Covariates with *P* value <0.25 during bivariate logistic regression analyses such as maternal education, wealth index, child's sex and age, food security status, antenatal care follow-up, birth interval, introduction of complementary foods, dietary diversity, meal frequency, undernutrition (stunting, underweight), and presence of fever and diarrhea were retained for multivariable logistic regression analysis to control for all possible confounders and to identify predictors of anemia. Multicollinearity between independent variables was checked using the value of standard error (SE), whereby all variables with SE less than 2 were considered. Model fitness was checked with the Hosmer–Lemeshow test and its *P* value was greater than 0.05. In a multivariable analysis, adjusted odds ratio (AOR) and 95% confidence interval were estimated to measure the strength of association between the dependent variable and covariates. The level of statistical significance was declared at *P* value <0.05.

### 2.7. Ethical Approval

Before the commencement of data collection, Haramaya University Institutional Health Research Ethics Review Committee (IHRERC) reviewed and approved the study with reference number C/Ac/R/D/01/878/18. Each study participant was informed, and voluntary, written, and signed consent was secured. Children who were found to be anemic during data collection were linked to the nearest health facility for treatment.

## 3. Results

### 3.1. Sociodemographic Characteristics

Of five hundred seventy-seven mothers/caregivers of infants and young children aged 6–23 months, a total of 531 participated in the study and making a response rate of 92.0%. Seven mother-child pairs were excluded according to the exclusion criteria and 39 were nonrespondents. The mean (±SD) age of the mother was 27 (±4) years. The majority, 467 (87.9%) mothers, had formal education. The mean (±SD) age of children was (14.7 ± 5.1) months. Slightly more than half, 271 (51%), of them were females. More than two-thirds, 356 (67%), of children were in the age range of 12–23 months (Table 1).

### 3.2. Feeding Practices

In our study, 526 (99.1%) infants and young children are ever breastfed. The majority, 482 (91.6%) children, initiated breastfeeding within one hour after delivery and 326 (61.4%) started complementary foods at 6 months. Nearly two-thirds, 348 (65.5%), and three-fifths, 324 (61%), achieved minimum meal frequency and minimum dietary diversity (≥4) food groups, respectively (Table 2).

### 3.3. Health-Care-Related Characteristics of Mothers

The majority, 519 (97.7%), mothers had at least one antenatal care (ANC) visit for the index child. Nearly all, 522 (98.3%), mothers delivered the index child at a health institution. All households had access to safe drinking water or pipe water (Table 3).

### 3.4. Prevalence of Malnutrition

**Figure 1:**
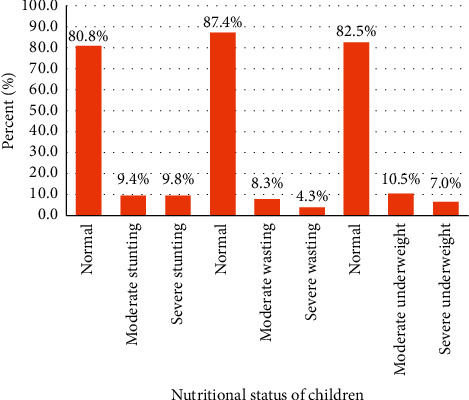
Nutritional status of children aged 6–23 months in Debre Berhan Town, North Shewa Zone, Ethiopia, 2018.

Of the 531 interviewed mother-child pairs, the prevalence of stunting was 19.2% (95 CI: 16.2–24.2), of which 9.8% was severely stunted. Similarly, the prevalence of wasting was 12.6% (95 CI: 9.8%–15.8%), of which 4.3% was severely wasted, while 17.5% (95% CI: 14.1%–21%) were underweight with 7% severely underweight (Figure 1).

### 3.5. Prevalence of Anemia

**Figure 2:**
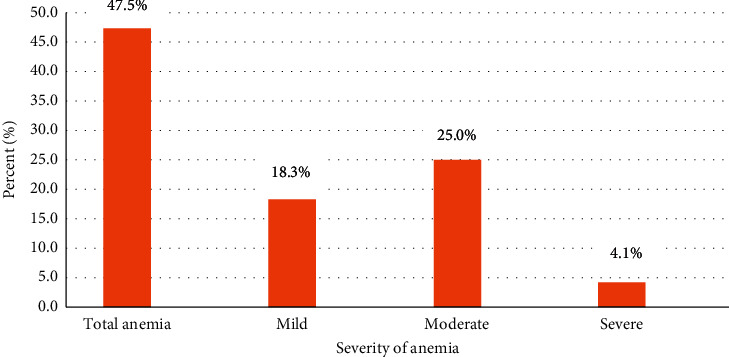
Prevalence and severity of anemia among infant and young children aged 6–23 months in Debre Berhan Town, North Shewa, Zone, Ethiopia, 2018.

In the present study, the mean (±SD) hemoglobin level was 10.8 (±1.87) g/dL with a maximum hemoglobin value of 16.8 g/dL and a minimum of 5.6 g/dL. The overall prevalence of anemia was 47.5% (252) (95% CI: 43.1%–51.4%) of which 18.3% (97) were mildly anemic, 25% (133) moderately anemic, and 4.1% (22) severely anemic (Figure 2).

The prevalence of anemia among children of mothers who had no education and had primary education was 64.1% and 66.1%, respectively, and the prevalence decreased as mothers/caregivers attained secondary education and above (36.4%). Anemia varies with the child's sex and age. More than half, 53.8% (140), of males and 41.3% (112) of females were anemic, but the prevalence decreased as the child age increased (Table 4).

### 3.6. Factors Associated with Anemia

In bivariable logistic regression analyses, maternal education, household's wealth index, child's sex and age, food security status, minimum dietary diversity, met minimum meal frequency, antenatal follow-up, history of diarrhea, fever two weeks prior to data collection, stunting, underweight, birth interval, and age at the commencement of complementary feeding were significantly associated with anemia.

In multivariable logistic regression analysis, food insecurity, ANC follow-up, minimum dietary diversity, met minimum meal frequency, underweight, and stunting were significantly associated with anemia.

Children from food-insecure households had 2.7 times (AOR = 2.7, 95% CI: 1.6–4.5) higher odds of being anemic than those children from food-secure households. The odds of anemia were reduced by 50% (AOR = 0.5, 95% CI: 0.3–0.9) among children born to mothers who had four or more ANC visits compared with children born to mothers who had fewer than four ANC visits. The odds of anemia were reduced by 75% among children who met their minimum meal frequency (AOR = 0.25, 95% CI: 0.14–0.45) compared with those children with unmet meal frequency. Similarly, children with less minimum dietary diversity were 2.5 times (AOR = 2.5, 95% CI: 1.4–4.3) more likely to be anemic than those children with achieved minimum dietary diversity. Stunted children were 2.3 times (AOR = 2.3, 95% CI: 1.2–4.3) more likely anemic, while the underweight children had 2.7 times (AOR = 2.7, 95% CI: 1.4–5.4) higher odds of anemia (Table 5).

## 4. Discussion

The present study assessed the prevalence of anemia and associated factors among infant young children aged 6–23 months. The World Health Assembly set a fifty percent reduction of anemia among pregnant women by the year 2025 as a global nutrition target. But the magnitude of anemia remains a significant public health concern across the globe [33]. Anemia is one of the severe public health issues, where 57% under-five children are anemic in Ethiopia [11].

The study findings showed that 47.5% of infants and young children aged 6–23 months were anemic. The magnitude is unacceptably high, which needs a strong effort to reduce where the level is not more a public health problem. This finding is similar to the studies reported in China (51.3%) [34] and Romania (46%) [35]. However, it is higher than the results of studies done in China (18.7%–36.6%) [36] and Brazil (29%) [37]. The possible reason for such variation could be the differences in socioeconomic and study period. On the other hand, the result of this study is lower than studies conducted in India (71.9%) [38], Egypt (66%) [39], Cote Devore (78.1%) [40], Kenya (73.2%) [41], and Ethiopia (66%) (53.7%) [21, 22], respectively. The possible differences between the current study and the aforementioned studies might be variations in the study setting, period, and geographical location. The variation might also be due to a difference in socioeconomic status, urbanization, place of residence, and child feeding practices of the participants.

In this study, there was a significant association between ANC follow-up and anemia. This is in line with a study done in Ethiopia [22]. Children born to mothers who had focused ANC follow-up had 50% fewer odds of being anemic than children of mothers who had fewer than four antenatal care visits. This is due to the fact that mothers who attend ANC may receive nutrition education and counseling about maternal and infant and young child feeding practices and care during ANC contacts [42]. One should recognize that an investment in maternal health services could also improve the health of their offspring, in which case anemia is one of the public health problems among under-five children and requires such an intervention.

Children from food-insecure households were 2.7 times more likely to be anemic compared to children in food-secure households. This is consistent with a study done in the US [43]. This could be explained by the fact that food-insecure households are less likely to access adequate, diversified, nutrient, and energy-dense diets. This finding implies that prevention of anemia among the target children requires giving full attention to the household's food security status.

Dietary diversity and meal frequency were also significantly associated with anemia. This is consistent with studies done in China [44] and Ethiopia [21, 22]. Children with less minimum dietary diversity were 2.5 times more likely to be anemic than children who used to eat ≥4 food groups. This might be due to the effect of cereal-based undiversified or monotonous complementary foods that lack essential minerals and vitamins (iron, vitamin A, vitamin B12, and folate) and thus is a major risk factor for anemia among infants and young children [45]. Similarly, children who met minimum meal frequency were 75% less likely to be anemic than those children with unmet minimum meal frequency. This might be due to the fact that children with high meal frequency are more likely to get enough amount of macronutrient, which ensures the child's optimal nutrition through the prevention of deficiency of essential nutrients. The findings related to dietary practices imply that there is a need to improve children's feeding practices to reduce the burden of anemia among the study population.

Moreover, undernutrition was found to be a significant predictor of anemia. This finding is consistent with the studies done in Rwanda [46] and northeast Ethiopia [21]. Stunted children were 2.3 times more likely to be anemic than their counterparts, and underweight children had 2.7 times higher odds of being anemic than nonunderweight children. This is due to the fact that undernourishment leads to both macronutrient and micronutrient deficiencies, such as protein, iron, and vitamin A, which are responsible for iron deficiency. This implies that prevention of anemia among infants and young children should take into account designing interventions that target the prevention of macronutrient undernutrition.

This study has the following limitations. First, due to the nature of the design, the study might not show causal relationships between predictors and anemia. Second, the study did not assess other risk factors for anemia such as intestinal parasites, HIV, genetic disorders, folate, vitamin B12, and vitamin A deficiencies. Third, the study used only hemoglobin level to define anemia, which does not reveal the body's level of iron stores. Fourth, household food security was assessed for the past four week's period. This could subject the study to recall and social desirability bias. Lastly, there could be possibilities for anthropometric and hemoglobin measurement errors, but all possible efforts were made to minimize all forms of errors. Despite all the aforementioned limitations, the study tried to assess the prevalence of anemia and associated factors among infants and young children aged 6–23 months at the population level. Nevertheless, the interpretation of the findings of the study needs to be carefully made as measurement error in anthropometry and hemoglobin level determination can either underestimate or overestimate the results of the study.

## 5. Conclusion

Based on the WHO cutoff point, the prevalence of anemia observed among children aged 6–23 months in the study area is a severe public health problem. Household food insecurity, less frequent antenatal care follow-up, dietary diversity, meal frequency, and undernutrition were significantly associated with anemia. Therefore, integrated efforts need to be made to improve infant and young appropriate feeding practices and maternal ANC service utilization. Similarly, multisectorial work needs to be emphasized to ensure household food security in the study community.

## Figures and Tables

**Table 1:** Sociodemographic characteristics of mothers/caregivers and infants and young children aged 6–23 months in Debre Berhan Town, North Shewa Zone, 2018 (*n* = 531).

Variables			Frequency	Percentage
Maternal age (years)	19–24		149	28.1
	25–29		253	47.6
	≥30		129	24.3

Maternal religion	Orthodox		505	95.1
	Muslim		15	2.8
	Others^*∗*^		11	2.1

Maternal ethnicity	Amhara		490	92.3
	Oromo		37	7
	Others^*∗∗*^		4	0.7
Maternal level of education	Have no formal education		34	6.4
	Able to read and write		30	5.6
	Grades 1–8		137	25.8
	Grades 9–12		155	29.2
	College and above		175	33

Maternal marital status	Single		19	3.6
	Currently married		500	94.2
	Divorced		7	1.3
	Widowed		5	0.9

Maternal occupation	Housewife		293	55.2
	Merchant		75	14.1
	Government employee		95	17.9
	Private employee		52	9.2
	Others^*∗∗∗*^		16	3

Husband educational status (*n* = 500)	Unable to read and write		6	1.2
	Able to read and write		16	3.2
	Grades 1–8		110	22
	Grades 9–12		162	32.4
	College and above		206	41.2

Husband occupation (*n* = 500)	Government employee		209	41.2
	Private employee		152	30.4
	Merchant		110	22
	Farmer		11	2.2
	Others^*∗∗∗∗*^		18	3.6

Family size	≤3		190	35.8
	4-5		273	51.4
	≥6		68	12.8

Number of under-five children	One		430	81
	2–3		101	19

Child age (in completed months)	6–11		175	33
	12–17		176	33.1
	18–23		180	33.9

Child health status	Any history of illness	Yes	141	26.6
		No	390	73.4
	Diarrhea	Yes	44	8.3
		No	487	91.7
	Fever	Yes	89	16.8
		No	442	83.2
	Vomiting	Yes	7	1.3
		No	524	98.7

Other^*∗*^: protestant = 10, self-believing = 1, other^*∗∗*^: Tigre = 2, Gurage = 2, other^*∗∗∗*^: daily labor = 11, farmer = 5, other^*∗∗∗∗*^: daily labor = 9, student = 3, guardian = 6.

**Table 2:** Distribution of selected IYCF indicators among infants and young children aged 6–23 months in Debre Berhan Town, North Shewa Zone, 2018.

Variables (*n* = 531)	Frequency	Percent
*Ever breastfed*		
Yes	526	99.1
No	5	0.9

*Timely initiation of breastfeeding (n* *=* *526)*		
Less than one hour	482	91.6
After one hour	44	8.4

*Currently breastfeed (n* *=* *526)*		
Yes	434	82.5
No	92	17.5

*Prelacteal feeding practice*		
Yes	9	1.7
No	522	97.3

*Bottle feeding*		
Yes	258	48.6
No	273	51.4

*Time of initiation of complementary food (520)*		
<6 months	80	15.1
At 6 months	326	61.4
>6 months	114	21.5

*Received MMF*		
Yes	348	65.5
No	183	34.5

*Received MDD*		
Yes	324	61
No	207	39

MMF: minimum meal frequency. MDD: minimum dietary diversity.

**Table 3:** Health care and environmental health-related characteristics of mothers or caregivers in Debre Berhan Town, North Showa, Zone, Ethiopia, 2018.

Variables (*n* = 531)	Number	Percent
*Birth interval (index child)*		
First birth	333	62.7
<24 months	81	15.3
≥24 months	117	22

*Antenatal care (ANC) follow-up*		
Yes	519	97.7
No	12	2.3

*Number of ANC visits (n* *=* *519)*		
<3	169	32.6
≥4	350	67.4

*Place of delivery*		
Home	9	1.7
Hospital	522	98.3

*PNC*		
Yes	205	38.6
No	326	61.4

*Availability of latrine*		
Yes	530	99.8
No	1	0.2

*Hand washing after toilet*		
Yes	527	99.2
No	4	0.8

**Table 4:** Prevalence of anemia among infant and young children aged 6–23 months by selected maternal and child characteristics in Debre Berhan Town, Ethiopia, 2018 (*n* = 531).

Variables	Anemia	
	Yes	No
	Number (%)	Number (%)
*Maternal education*		
Have no formal education	41 (64.1)	23 (35.9)
Primary education	91 (66.4)	46 (33.6)
Secondary and above	120 (36.4)	210 (63.6)

*Maternal occupation*		
House wife	153 (52.2)	140 (47.8)
Merchant	44 (58.7)	31 (41.3)
Private employee	15 (28.8)	37 (71.2)
Government employee	32 (33.7)	63 (66.3)
Others	8 (50)	8 (50)

*Family size*		
≤3	92 (48.4)	98 (51.6)
4–5	126 (46.2)	147 (53.8)
≥6	34 (50)	34 (50)

*Household's wealth index*		
Lowest quantiles	59 (55.7)	47 (44.3)
Second quantiles	57 (55.9)	45 (44.1)
Middle quantiles	50 (53.2)	44 (46.8)
Fourth quantiles	53 (39.3)	82 (60.7)
Highest quantiles	33 (35.1)	61 (64.9)

*Child's sex*		
Male	140 (53.8)	120 (46.2)
Female	112 (41.3)	159 (58.7)

*Number of under-five children in households*		
Only one	197 (45.8)	233 (54.2)
2–3	55 (54.5)	46 (45.5)

*Age of infants and young children (in months)*		
6–8	40 (52.6)	36 (47.2)
9–11	66 (66.7)	33 (33.3)
12–17	78 (44.3)	98 (55.7)
18–23	68 (37.8)	112 (62.2)

*Food security status*		
Food-insecure	170 (65.9)	88 (34.1)
Food-secure	82 (30)	191 (70)

*ANC follow-up*		
≥4 visits	119 (34)	231 (66)
<4 visits	125 (74)	44 (26)

*Birth interval*		
First birth	155 (46.5)	178 (53.5)
<24 months	55 (67.9)	26 (32.1)
≥24 months	42 (35.9)	75 (64.1)

*Exclusive breastfeeding*		
Yes	178 (42)	246 (58)
No	70 (68.6)	32 (31.4)

*Introduction of complementary foods*		
<6 months	50 (62.5)	30 (37.5)
>6 months	78 (68.4)	36 (31.6)
At 6 months	120 (36.8)	206 (63.2)

*Bottle feeding*		
Yes	137 (53.1)	121 (46.9)
No	115 (42.1)	158 (57.9)

*Minimum meal frequency*		
Yes	111 (31.9)	237 (68.1)
No	141 (77)	42 (23)

*Minimum dietary diversity*		
<4 food groups	147 (71)	60 (29)
≥4 food groups	252 (47.5)	279 (52.5)

*Length for age*		
Normal	185 (43.1)	244 (56.9)
Stunted	67 (65.7)	35 (34.3)

*Weight for age*		
Normal	194 (44.3)	244 (56.7)
Underweight	58 (62.4)	35 (37.6)

*Weight for height*		
Normal	36 (57.7)	31 (46.3)
Wasted	216 (46.6)	248 (53.4)

*History of fever*		
Yes	57 (64)	32 (34)
No	195 (44.1)	247 (55.9)

*History of diarrhea*		
Yes	34 (77.3)	10 (22.7)
No	218 (44.8)	269 (55.2)

**Table 5:** Factors associated with anemia among infant and young children aged 6–23 months in Debre Berhan Town, Ethiopia, 2018 (*n* = 531).

Variables	Anemia		COR (95% CI)	*P* value	AOR (95% CI)
	Yes	No			
*Maternal education*					
No formal education	41 (64.1)	23 (35.9)	3.1 (1.8, 5.5)	0.001	0.5 (0.2, 1.2)
Primary education	91 (66.4)	46 (33.6)	3.5 (2.3, 5.3)	0.001	1.0 (0.5, 1.8)
Secondary and above	120 (36.4)	210 (63.6)	Reference		Reference

*Wealth index*					
Lowest quantiles	59 (55.7)	47 (44.3)	2.32 (1.3, 4.1)	0.004	0.8 (0.4, 1.8)
Second quantiles	57 (55.9)	45 (44.1)	2.34 (1.3, 4.1)	0.004	0.9 (0.4, 1.9)
Middle quantiles	50 (53.2)	44 (46.8)	2.1 (1.2, 3.8)	0.13	1.1 (0.5, 2.3)
Fourth quantiles	53 (39.3)	82 (60.7)	1.2 (0.7, 2.0)	0.523	0.9 (0.5, 1.8)
Highest quantiles	33 (35.1)	61 (64.9)	Reference		Reference

*Child's sex*					
Male	140 (53.8)	120 (46.2)	1.7 (1.2, 2.3)	0.004	1.0 (0.6, 1.6)
Female	112 (41.3)	159 (58.7)	Reference		Reference

*Age of child*					
6–8 months	40 (52.6)	36 (47.4)	1.8 (1.0, 3.1)	0.29	0.6 (0.3, 1.3)
9–11 months	66 (66.7)	33 (33.3)	3.3 (1.9, 5.5)	0.0001	1.5 (0.7, 3.0)
12–17 months	78 (44.3)	98 (55.7)	1.3 (0.8, 2.0)	0.21	1.0 (0.6, 1.7)
18–23 months	68 (37.8)	112 (62.2)	Reference		Reference

*HH food security status*					
Food-insecure	170 (65.9)	88 (34.1)	4.5 (3.1, 6.5)	0.0001	2.7 (1.6, 4.5)^*∗∗*^
Food-secure	82 (30)	191 (70)	Reference		Reference

*ANC follow-up*					
≥4 visits	119 (34)	231 (66)	0.2 (0.1, 0.3)	0.0001	0.5 (0.3, 0.9)^*∗*^
<4 visits	133 (73.5)	48 (26.5)	Reference		Reference

*Birth interval*					
First birth	155 (46.5)	178 (53.5)	1.5 (1.0, 2.4)	0.047	1.14 (0.7, 2.0)
<24 months	55 (67.9)	26 (32.1)	3.8 (2.1, 6.9)	0.0001	2.0 (0.9, 4.4)
≥24 months	42 (35.9)	75 (64.1)	Reference		Reference

*Introduction of complementary foods*					
<6 months	50 (62)	30 (37.5)	2.9 (1.7, 4.7)	0.0001	0.9 (0.4, 1.9)
>6 months	78 (68.4)	36 (31.6)	3.7 (2.4, 5.9)	0.0001	1.5 (0.8, 2.8)
At 6 months	120 (36.8)	206 (63.2)	Reference		Reference

*Met MMF*					
Yes	111(31.9)	237 (68.1)	0.14 (0.1, 0.2)	0.0001	0.25 (0.14, 0.4 5)^*∗∗*^
No	141 (77)	42 (23)	Reference		Reference

*MDD*					
<4 food groups	147 (71)	60 (29)	5.1 (3.5, 7.5)	0.0001	2.5 (1.4, 4.3)^*∗∗*^
≥4 food groups	105 (32.4)	219 (67.6)	Reference		Reference

*Length for age*					
Stunted	67 (65.7)	35 (43.1)	2.5 (1.6, 3.9)	0.0001	2.3 (1.2, 4.3)^*∗*^
Not stunted	185 (43.1)	244 (56.9)	Reference		Reference

*Weight for age*					
Underweight	58 (62.4)	35 (37.6)	2.1 (1.3, 3.3)	0.002	2.7 (1.4, 5.4)^*∗∗*^
Normal	194 (44.3)	244 (55.7)	Reference		Reference

*Fever*					
Yes	57 (64)	32 (36)	2.3 (1.4, 3.6)	0.001	1.0 (0.5, 1.9)
No	195 (44.1)	247 (55.9)	Reference		Reference

*Diarrhea*					
Yes	34 (77.3)	10 (22.7)	4.2 (2.0, 8.7)	0.0001	1.1 (0.4, 2.8)
No	218 (44.8)	269 (55.2)	Reference		Reference

^*∗*^ *P* value <0.05, ^*∗∗*^*P* value< 0.005.

## Data Availability

The data used to support the findings of this study are available from the corresponding author upon request.

## Supplementary Materials

Supplementary Materials Supplementary Materials. Supplementary File 1: laboratory procedure and interpretation protocol.
