# Supplementary material for: Prevalence of Anemia and Associated Factors among Infants and Young Children Aged 6–23 Months in Debre Berhan Town, North Shewa, Ethiopia
Source: J Nutr Metab. 2020 Dec 17;2020:2956129. doi: 10.1155/2020/2956129 (PMC7768586; doi:10.1155/2020/2956129)
Supplement: Supplementary Materials — Supplementary File 1: laboratory procedure and interpretation protocol. [file 2956129.f1.docx]

**Laboratory procedure and interpretation**

|  | Assemble Materials on workspace |
| --- | --- |
|  | Place gloves on |
|  | Obtain HemoCue Hb 301+  Confirm:   - Hemocue Hb 301 Micro cuvettes Lot # and expiry date. |
|  | Turn HemoCue HB 301+ ON   - Press and hold the left button to activate the display (ON/OFF) - Display shows the version number of the program - All symbols appear on the display. - The analyzer verifies the optic and electronic performance. - Hemocue displays previous result. |
|  | Prepare Hemocue for loading a microcuvette   - Pull the micro cuvette holder out - After 10 seconds the display will show three flashing dashes and the Hemoglobin symbol - Indicates that self-test is passed. - If the Self-Test fails note error code. Do not proceed testing. - When an error code was displayed due to self-test failure, quality control measure was performed according to the recommended guideline |
|  | Take HemoCue Microcuvette. Open packaging just before use.   - keep microcuvette at temperature ranges (15-30oC) - Confirm Lot # and Expiry date. - Hold microcuvette opposite to fill end. - Place on clean working surface. |
|  | Use appropriate capillary skin puncture device. |
|  | Perform capillary skin puncture   - Patient’s hand is warm and relaxed. - Use only the middle three fingers. - Clean the finger with disinfectant. - Allow to air dry. - Place skin puncture device on the side of the fingertip, not in the center. - Apply slight pressure to tip of finger and make the puncture. - Wipe away the first 2 drops of blood. - Re-apply light pressure towards the fingertip until another drop of blood is formed. |
|  | Filling the microcuvette with sample   - When blood drop is large enough, fill the microcuvette in one continuous process. - DO NOT REFILL. - Wipe off excess blood from outside of the micrcuvette. - Use lint free tissue. - Be careful not to touch the open end of the microcuvette which will result in blood being drawn out of the microcuvette. - Look for air bubbles in the filled microcuvette, if present, discard. |
|  | Hemoglobin measurement.   - Place the filled microcuvette in the cuvette holder. - Gently slide the cuvette holder to the measuring position. - During the measurement the hour glass symbol will display. |
|  | Hemoglobin Result   - Hemoglobin value is displayed after 15-60 seconds. - The result will remain on the display as long as the cuvette holder is in the measuring position. - Do Not open the cuvette holder until the result is documented - If using batteries, the analyzer will automatically turn off after 5 minutes   Note: If the analyzer turns off automatically – turn the analyzer ON – the result displayed would be the current participant result. |
|  | Hemoglobin Result Recording   - On the participant Record Sheet include: - Hemoglobin value (g/L) - Date/time performed. - Operator ID |
|  | Hemoglobin Result Review  Hemocue Hb 301+ expected values (as per manufacturer guidelines):   - Mild anemia = 10.0-10.9 g/dl for children under age 5 and pregnant women and 10.0-11.9 g/dl for non-pregnant women. - Moderate anemia = 7.0-9.9 g/dl, - Severe anemia <7.0 g/dl - Repeat any unexpected values or critical values. |
|  | Discard Microcuvette   - Appropriate waste container for infection control. - Clean cuvette holder if contamination is visible with disinfectant wipe. - Cuvette holder is cleaned after each day of use. |
